# Comment on “Computed Tomography Evaluation of Craniomandibular Articulation in Class II Division 1 Malocclusion and Class I Normal Occlusion Subjects in North Indian Population”

**DOI:** 10.1155/2013/932701

**Published:** 2013-05-08

**Authors:** Ashutosh Dixit, Ridhima Birmani Gaunkar, Varun Arora, Seema K. Dixit, Narendra Kumar Gupta, Pratik Chandra, Bhaskar Agarwal

**Affiliations:** ^1^Department of Periodontics, Seema Dental College, Rishikesh 249203, India; ^2^Community Dentistry, Department of Public Health Dentistry, Goa Dental College, Goa 403401, India; ^3^Active Research Group, Arun Professional Services, Tulsidas Marg, Chowk, Lucknow 226003, India; ^4^Department of Endodontics & Conservative Dentistry, Seema Dental College, Rishikesh 249203, India; ^5^Department of Prosthodontics, Babu Banarasi Das College of Dentistry, BBD University, Lucknow 227015, India; ^6^Department of Orthodontics, Saraswati Dental College and Hospital, Lucknow 227105, India; ^7^Department of Prosthodontics, Faculty of Dental Sciences, King George Medical University, Lucknow 226003, India

## Abstract

Clear statement of objective, appropriate location of landmarks and removal of subjective bias in measurement is essential in all kinds of research, especially, orthodontics. The research design should be rationalistic, purposeful, and in accordance with the objectives of the study. In this communication, we highlight the errors in research design, measurement, analysis, and inferences drawn with the help of a published article as the primary source to explain these simple but useful points.

## 1. Unclear Objective

The authors state the purpose of study is “to investigate the Craniomandibular articulation morphology and position of condyle in mandibular fossae in Angle's class I normal occlusion and Angle's class II division 1 malocclusion”; however, no objective attempt has been made to establish distinction between two classes, and in the absence of any such distinction between two classes, it is difficult to assume that the articulation morphology claimed to be of a particular class is a true representation of that particular class and not the other class. It is disappointing to see that, despite using quantitative tools, the authors have tried to make only “within-class comparisons” and no attempt has been made to compare the morphological parameters “between two classes” to differentiate and establish any finding to be representative of a particular class. We fail to understand if the purpose of the study was not to differentiate between two occlusal classes, then why the entire study has been carried out taking two occlusal classes separately. In our view, if the purpose of study was to compare between left and right sides, then there was no need to differentiate between two classes. Moreover, if this was the purpose, then a combined assessment of two classes would have given a better ground for differentiation owing to enhanced sample size. 

## 2. Statistical Tools

There is a lot of confusion and a number of errors while stating and using the statistical tools. The authors have measured different morphological parameters using a continuous scale. The basis of their measurements happens to be based on subjective interpretation/tracings. The authors have mentioned using two examiners to rule out subjective error in measurement. However, they have mentioned to have used K-score for assessment of interexaminer reliability; it seems that authors are referring here to kappa-statistic. To the best of our knowledge, Kappa-statistic is used to compare the ordinal data or ordinal transformation of continuous data [1]. For continuous data, paired “*t*”-test, Pearson correlation, Bland and Altman coefficient, Lin's concordance coefficient, and BSI coefficient of repeatability are some of the valid choices widely used for assessing interexaminer reliability of orthodontic measurements [2, 3]. 

The authors also seem to be confused in determining the statistical plan of the study. They mention *“Each measurement was compared by two-factor analysis of variance (ANOVA), and the significance of mean difference was done by Newman-Keuls post hoc test to evaluate the average of differences between left and right side for each element of the sample, while change in AJS and PJS was done by paired t test. A two-tailed *(*α* = 2)* probability *(*P*)* less than 0.05 *(*P* < 0.05)* was considered statistically significant”* in Section 2; however, nowhere in the paper we get use of any two-factor analysis of variance (ANOVA). In Table  1 of their paper, which though does not mention the type of statistical test used, yet as the comparisons are made for a single factor and between two sides (left and right), neither use of two-factor analysis nor ANOVA seems to be a justified choice. 

## 3. Rationale of Finding Difference between Anterior and Posterior Joint Space Measurements

 We do not understand any applied utility of measuring difference between anterior and posterior joint space measurements as they are two different entities and measuremental variation in them could be possible unless they are studied in context with type of occlusal class. As the authors have made no attempt to make such differentiation, hence, it remains unexplained. 

## 4. Discussion

The authors mention that the findings of their study “*could be a valuable reference for evaluation and comparison of TMJ morphology in different malocclusions in north Indian population*;” however, in the absence of any attempt to differentiate between Class I and Class II occlusal types, it is difficult to assume that these measurements would serve any purpose as a reference data for *different malocclusions*.

Use of explanatory comment “*Since the sagittal evaluation showed no significant differences regarding condylar dimension and positioning, the asymmetry in the posterior articular space can be explained by different dimensions of mandibular fossae*” does not hold ground unless it has been substantiated by the evidence which has not been provided anywhere in the study.

Similarly, the explanatory comment *“Result of our finding regarding the concentric positioning of condyle is*…*preference during the mastication*” remains unexplained as to why *left side* is affected in malocclusion. To the best of our knowledge, no such side specific condylar position in malocclusion cases is established anywhere in the literature.

In view of the above discrepancies, the scope of the study is limited and could have been widened if a comparative assessment of Class I and II occlusion classes was done. The purpose of the study as stated by the authors to provide reference values for class I and II occlusions could also have been fulfilled with a changed plan of analysis.
